# Supplementary material for: Glutamate Dehydrogenase Functions in Glutamic Acid Metabolism and Stress Resistance in Pyropia haitanensis
Source: Molecules. 2021 Nov 10;26(22):6793. doi: 10.3390/molecules26226793 (PMC8623670; doi:10.3390/molecules26226793)
Supplement: Supplementary file 1 [file molecules-26-06793-s001.zip › molecules-1457082-supplementary.pdf]

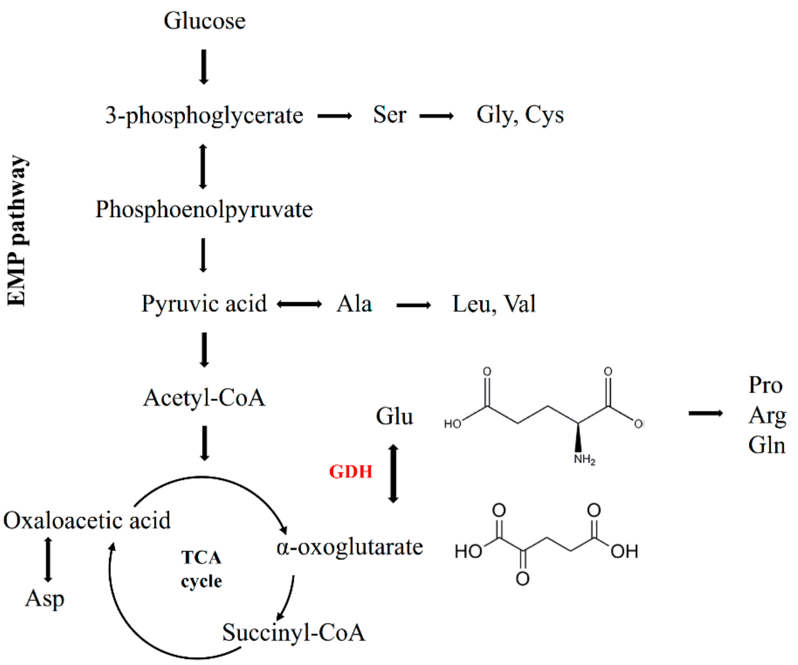

Figure S1. The glutamic acid biosynthetic pathway adapted from Kyoto Encyclopedia of Genes and Genomes (KEGG) pathway map01230. Abbreviations amino acid: Ser: serine; Gly: glycine; Cys: cystine; Ala: alanine; Leu: leucine; Val: valine, Glu: glutamic acid; Pro: proline; Arg: arginine; Gln: Glutarnine; Asp: Aspartic acid.

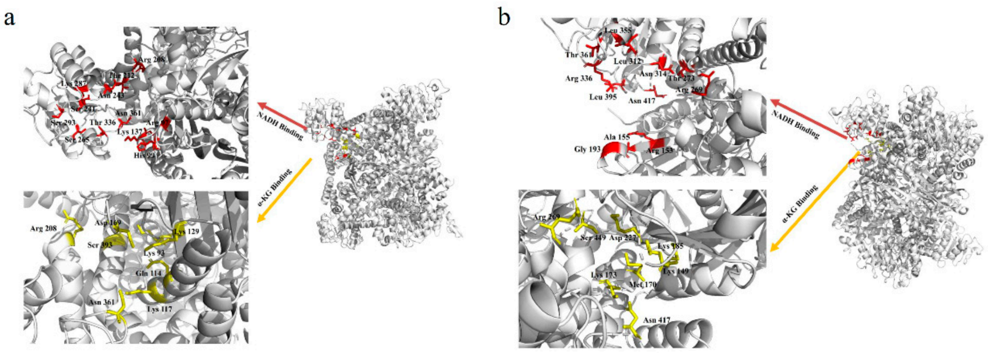

Figure S2. The predicted tertiary structures of PhGDH1 (**a**) and PhGDH2 (**b**). The presumed cofactor-binding sites are marked in red while the presumed substrate-binding sites in yellow.

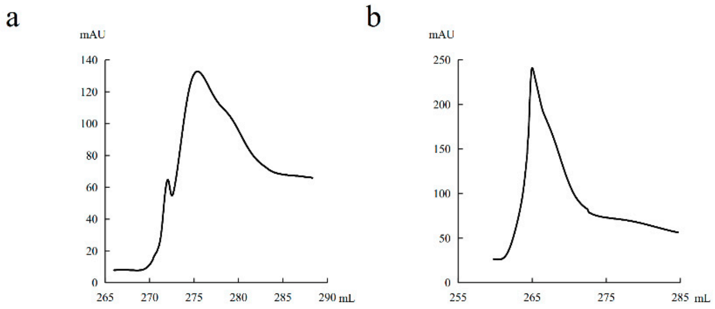

Figure S3. Purification of the recombinant PhGDHs. (**a**) The eluting peak of PhGDH1. (**b**) The eluting peak of PhGDH2.

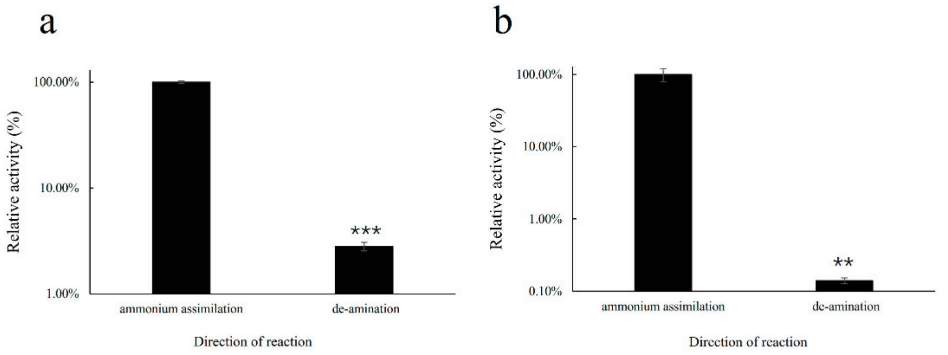

Figure S4. Comparisons of the relative activities between the direction of ammonium decomposition and ammonium assimilation for PhGDH1 (**a**) and PhGDH2 (**b**). *P < 0.05, **P < 0.01, and ***P < 0.001.

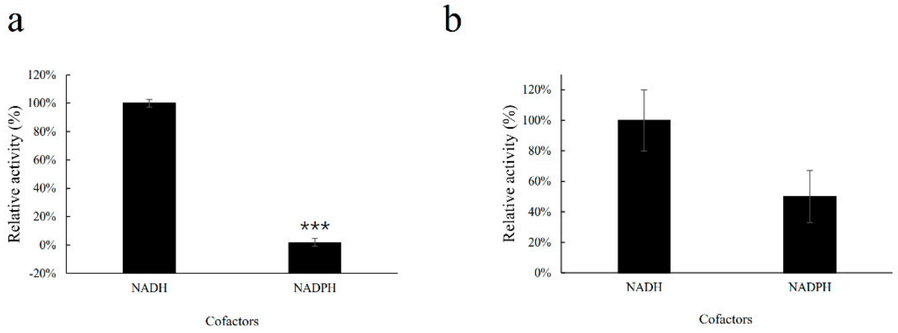

Figure S5. Relative activity of PhGDH1(**a**) and PhGDH2 (**b**) using the two cofactors. *P < 0.05, **P < 0.01, and ***P < 0.001.

Table S1. The K_{cat} values as well as K_m, V_m and K_{cat}/K_m of the PhGDH1/PhGDH2.

	substrate	K_m (mM)	V_m (μmol·min^{-1}·mg^{-1})	K_{cat} (S^{-1})	K_{cat}/K_m (S^{-1}·mM^{-1})
PhGDH1	NADH	0.12	1.3	1.52	12.67
	$(NH_4)_2SO_4$	4.99	0.65	0.76	6.33
	α-oxoglutarate	0.16	0.65	0.76	6.33
PhGDH2	NADH	0.02	0.39	0.38	3.17
	$(NH_4)_2SO_4$	3.98	0.32	0.31	2.58
	α-oxoglutarate	0.104	0.32	0.31	2.58

Table S2. The RPKM values of glutamine synthetase. PT: Putian (Fujian Province), YC: Yancheng (Jiangsu Province). 1, 2, and 3 indicate samples collected in October, November, and December, respectively.

	Unigene0045080	Unigene0046035	Unigene0050875
PT1	0.073	0.393	0.11
PT2	0.11	0.063	0
PT3	0.253	0.19	0.063
YC1	0	0	0.11
YC2	0.07	0.067	0
YC3	0	0.13	0

Table S3. The variable-to-maximum fluorescence (F_v/F_m = (F_m-F_0)/F_m) was measured before abiotic stresses.

	**Area 1**	**Area 2**	**Area 3**
Size [pixels]	11001	5926	4595
F_0	115.22	95.01	124.92
F_m	370.18	286.21	317.43
F_v	254.97	191.2	192.51
QY_max (F_v/F_m)	0.69	0.67	0.56

Table S4. The coding sequences of *PhGDH*1/*PhGDH*2.

PhGDH1

ATGTCGGGCCTGTCGGCCAAGCTGCAGCCGGTGTTTGACGCGATCAAGGCACGCAACTCCAACGAGCCCGAGTTCCTCCAGGCCGCTGAAG
AGATTCTCCACTCGTTGGGTCCCGTCATCGAGGCTGACGGCGGTGACAAGTACATTCCGGTCACCAAGGCTCTGTTTGAGCCGGAGCGTGTC
ATCCAGTTCCGCGTTGCCTGGTACGATGACGACGGGGAGCTGCAAGTCAACCGTGGCTTCCGGGTGCAAATGAACTCGGCTATCGGGCCGT
ACAAGGGCGGGCTTCGGCTCCATCCCACTGTCAACCTCTCTATCCTCAAGTTCCTCGCAACGGAGCAGGTGCTGAAGAACGCGGTGACGAC
TCTGCCGCTGGGCGGTGGCAAGGGCGGGTCGGACTTCAACCCCAAGGGACGCTCTGAGGCGGAGGTGCGTCGCTTTTGCCAGGCCTTTATG
GGTGCTCTGGCTCGCCACATTGGTCCCGACACGGATGTTCCTGCTGGTGACATTGGCGTGTCCGGCCGGGAGATTGGCTACCTGTTTGGAGC
GTACAAGCGCGCGACCAACCGGTTTGAGGGCATCCTCACCGGGAAGGGCCCGACGTTTGGGGGCTCCCTCATCCGGCCCGAGTCCACGGGC
TACGGCGTGGTCATGTTTGTGCGTGAGATGCTCAAGGTCAAGGGCGATACGCTGGAAGGCAAGGTGGTCATCATTTCCGGCTCTGGCAACGT
AGCGCAGTACGCGACGGAGAAGGTGAACGCGCTGGGCGGCAAGGTCATTGCGTTGTCCGACTCGAAGGGCGTCCTCGTTGATGAGGAGGG
CATCACGGACAAGAAGCTGGCCGAGGTCATGGACATCAAGAACGTCCGCCGCACGTCGCTCAAGGCGTACACGGAGAAGTTTTCGAGTGC
CAAGTGGACGGACTTTTCCGAGCAGTACACGGACGAGGATAAGAAGTCAAACCCACTGTGGGAGGTCAAGTGTGACGTGGCTCTGCCGTGC
GCCACGCAGAACGAGCTCAACGCGGCCGGCGCCCAGGCGCTGGTGGACAATGGTGTCACGATTGTTGCGGAGGGGGCCAACATGCCCTCG
ACGCCGGGTGCCATCGAGGTGCTGCGCTCGGCCAAGACTGCATTTGCGCCTGGCAAGGCGAGCAACGCCGGTGGGGTGGCCACGAGCGGA
CTCGAGATGTCGCAAAACTCGCAGCGCATGGCGTGGTCTGCGGAGAAGGTGGAGGAGGAGCTGGATGGGATCATGACCCGCATCCATGCC
GACTGCGTGAAGACGGCCAAGGCGTACGACCGGGCTGGGGACTATGTGTTTGGCGCCAACGTGACGGGCTTCCTCAAGGTTGCTGAGGCTA
TGCTGGCGCAGGGGCTTGCCTGA

PhGDH2

ATGGCGACGCGGGCAGGGGGGGTTCCCGCCGCCGCTCCCCCCACCGCCGCTCCTGCCCCCGCCGTCGCTGCTGCTGCTGGCGTGGCGGGTCT
CGCCTCCCTGTCACCCCCCCCACGGTCCGTGCGGGCACGCCGCCCGGCGGTTGCCGCCGCTGGCGCGGCGGCCGCCGCGTCCACCACCACT
GCGAGCGGCGGTCCGCCGCCGCCGACGTCGCCCGGGGCGGCGGCCAAAGGCGAGCCAGAAGGGGGCACCTTCCTCGAGGGCGTCGACGCC
AACTTTCGGGCGGCGAGCGCGCACCTGCGGTCGGTGGACCCCGCGCTGCTGGAGAATATCCGCCGGTGCAACAGCACGACCGAGTTTGCCT
TTCCGCTCAAGCGGGACGACGGGAGCATCACCACCCTCACCGGCTACCGGGCGGTGCATTCGTACCATATGACGCCGTCCAAGGGCGGCAT
CCGCTACGCGCCGTCCGTGTCGCGGTCCGAGGTGGAGGCGCTCGCATGCCTGATGACGCTCAAGTGTGCGGTCGTGGAGGTGCCGTACGGG
GGGGCCAAGGGGGGGGTGGCCATCAACCGCGGTGACTACTCGCCGGCAGAGGTGGAGCGCATCACCCGCCGCTTCACGACTGAGCTGTAC
CGGCGCAACCTGATCGGCCCGGCCGTGGACGTGCCGGCGCCCGACTTTGGCACCACCGCGGGGGACATGGCGCACATTAAGGATACGTAC
ATGCAGCTGGAGGGCAAGTCGATGTTTGGGGCGGCGGCGGTGACGGGCAAGCCCGTCAGCCAGGGGGGCATCCGCGGGCGGGAGGAGGC
CACCGGCCTGGGCGTCTACTTTGGCGTCCGCGAGTTCCTGTCCGACCCGTCGGTCGCGGCGGCCGCGGGGCTGCCCACCCCGTGGAGCGTCC
CAAAGTCGACGTTTGCCATCCAGGGGCTTGGCAACGTCGGCTACTGGGCCGCCCACTTTATTGCCAAGAATGGCGGCCTCATCACGGCCGTC
GGCGAGCGGGACGGGACCGTGTCGGACCCGGCGGGTGTGGACGTGGAGGCCCTCAAGGTGCACCTGACCACTAATGGTGGCACGGTGGTC
GGCTTTACCAATGGCGGCAGCCCGTCGCTGTCGGTGATGCCCGACCCGTCGCTCGTCTTGTCTGCCGACGTGGACGTGCTCATCCCGGCGGC
GCTGGAAGGCGCCATCACGACCGCCAACGCGCGGGCGGTGCGCGCCAAGGTGGTCGCCGAGGCCGCCAATGGCCCCGTCACTGCTGGCGC
GGACGTGGTACTCGCCTCCGCCGGCGTGGTGGTCCTCCCCGACCTGGTCATGAACGCCGGCGGCGTGACGGTGAGCTACTTTGAGGTGGCCA
AGAACTTGGCGGGCCTCCGCTTTGGGCGGCTGACCCAGCGCGCGGAGGAGGCGGCGATGGAGAACCTGCTCGCGACACTGCAGACCCACG

GCGTCACCATCACCGACCGCGACCGCCGGCGGCTGATCATTGGCGCCGACGAGCGCGCGCACGTGTACAGCGGGCTGGAGGACAGCATGT GCGCGGCGTGTGGGGAGACGGTCAAGGTGGCCGCGGAGCTGGGCGTGTCGCTCCGCATCGCCGCCTACTACACCGCCATCCGGCGGGTGGC CGACACGTTTGAGAGCCGCGGGCTGTGGCCTTGA

Table S5. Primer required for experiment.

Name	Forward primer	Reverse primer
PhGDH1	5′-CCGGAATTCATGTCGGGCCTGTCGGCCAAGCTGC-3′	5′-CCCAAGCTTTCAGGCAAGCCCCTGCGCCAGCATA-3′
PhGDH2	5′-CCGGAATTCATGGCGACGCGGGCAGGGGGGGTTC-3′	5′-CCCAAGCTTTCAAGGCCACAGCCCGCGGCTCTCA-3′
K137D	5′-TCAACCCCGATGGACGCTCTGAGGCGGAGGTGCG-3′	5′-AGCGTCCATCGGGGTTGAAGTCCGACCCGCCCTT-3′
S293D	5′-GCCGCACGGATCTCAAGGCGTACACGGAGAAGTT-3′	5′-CCTTGAGATCCGTGCGGCGGACGTTCTTGATGTC-3′
G193D	5′-TCAACCGCGATGACTACTCGCCGGCAGAGGTGGA-3′	5′-AGTAGTCATCGCGGTTGATGGCCACCCCCCCCTT-3′
T361D	5′-ATGGTGGCGATGTGGTCGGCTTTACCAATGGCGG-3′	5′-CGACCACATCGCCACCATTAGTGGTCAGGTGCAC-3′
qPhGDH1	5′-GATTCTCCACTCGTTGGGTC-3′	5′-CCCGATAGCCGAGTTCATTT-3′
qPhGDH2	5′-GAGTTTGCCTTTCCGCTCAA-3′	5′-GCACACTTGAGCGTCATCA-3′
EF2	5′-CTGTCCAAGAGTGCGAACAAG-3′	5′-CTCGTCCATGCCATACTCGT-3′
